# Distribution and prevalence of oral mucosal lesions in residents of old age homes in Delhi, India

**DOI:** 10.3126/nje.v8i2.18708

**Published:** 2018-06-30

**Authors:** Nisha Rani Yadav, Meena Jain, Ankur Sharma, Roma Yadav, Meetika Pahuja, Vishal Jain

**Affiliations:** 1 Senior lecturer, Department of Public Health Dentistry, Manav Rachna Dental College, Faridabad, Haryana, India.; 2 Reader, Department of Public Health Dentistry, Manav Rachna Dental College, Faridabad, Haryana, India.; 3 Lecturer, Department of Public Health Dentistry, Manav Rachna Dental College, Faridabad, Haryana, India.; 4 Senior Lecturer, Department of Public Health Dentistry, NIIMS Dental College, Jaipur, Rajasthan, India.; 5 Senior Lecturer, Department of Public Health Dentistry, Subharti Dental College, Meerut, U.P, India.; 6 Private practitioner, Faridabad

**Keywords:** prevalence, geriatric dentistry, oral lesions

## Abstract

**Background:**

It has been seen that very less attention has been given to the oral health of the geriatric population residing in old age homes and as the oral mucosal lesions are a matter of concern for this growing population. Therefore, a study was done with the objective of finding the prevalence of oral mucosal lesions and the distribution of oral mucosal lesions among 65-74 year old residents of old age homes in Delhi, India.

**Materials and Methods:**

A cross sectional study was done on 65-74 year old age group elders of old age homes in Delhi. A total of 464 subjects participated in the study. Oral Health Assessment Form, WHO was used for assessing oral mucosa. Clinical examination was performed using two mouth mirrors under natural illumination in a systematic manner. Data was processed and analyzed using SPSS version 23.

**Results:**

Out of a total of 464 subjects, 291 (62.70%) were males and 173 (37.30%) were females. Oral mucosal lesions seen in the study subjects were malignant tumours, leukoplakia, lichen planus, ulcerations, ANUG, Abscess and candidiasis. Leukoplakia was seen in 70 subjects (15%) and was present on buccal mucosa in the majority. A malignant tumour was seen in 7 subjects (1.5%) and commonly seen area is floor of mouth.

**Conclusion:**

Prevalence of oral mucosal lesions among residents of old age homes shows the need for increased preventive and diagnostic measures for prevention and early identification of oro-mucosal lesions. Taking adequate care for oro-mucosal health of elderly people residing in old age homes is necessary.

## Introduction

There has been a renewed interest in study of geriatric oral health owing to a recent increase in this population [1]. Oral-mucosal lesions, along with dental caries and periodontal diseases are often subject of concern among elderly [2]. Oral mucosal lesions (OMLs) are usually associated with various local and/or systemic conditions. Thus, it has become an intriguing subject in the recent literature.

Oral mucosal health is directly related to the general health. It performs many functions like protection, sensation and secretion. With increasing age, it gets thinner and even the collagen synthesis by connective tissue gets slower, which further makes the mucosa less resistant to cancer causing agents and other toxic substances. Ultimately, this leads to cancer in some individuals [2]. So, age is an important contributing factor for oral mucosal lesions.

A study conducted in Iran in institutionalized elderly patients observed oral OMLs in 98% of the elder individuals [3].OMLs have been reported in 41.2% of the Indian population [4].The part of oral mucosa which can be involved is the right and left buccal mucosa, followed by labial mucosa, tongue, gingiva, hard/soft palate, and alveolar mucosa [5]. Oral mucosal lesions interfere with the eating, drinking and speaking practices of the patient. So the routine activities of the person get hampered, as the lesion cause pain and discomfort [6]. As has been established by the WHO (2013), a population ageing more than 60 years should be considered to be an elderly population [7].

The latest national census conducted in India in 2011 presented that 8.6% of the population were 60 years or more [8] indicating that the health of geriatric population is important. The various barriers in the maintenance of oral health in elderly people have been recognized. These include the lack of trained health workers, incapability of caretakers or patients to maintain good oral hygiene, deficit financial support, and inefficient dental health care delivery structure [9].

Till now little has been done for this geriatric population in the country. However, before planning any local or national oral health program; data regarding the extent of the problem have to be collected.

The recent, 2011 census showed that Delhi, the capital of India, inhabits 16.7 million population making it second largest metropolis in the country [8]. Therefore, a study was planned to assess the prevalence of oral mucosal lesions among elderly residing in old age homes of Delhi, India.

## Methodology

### Study design and participants

A cross sectional study was conducted among 65-74 year old residing in old age homes in Delhi, India. Data collection was performed during October-November 2017.

### Sample size calculation

Prior to the main study, a pilot study was conducted among 30 elders of an old age home, which were excluded from the final sample. This old age home was not a part of the study sample. Considering the prevalence of oro-mucosal lesion (52%) obtained from the pilot study and a non-response rate of 15%, a minimum sample size of 460 subjects was estimated.

Delhi is divided into 5 different regions with a total of 38 old age homes, according to the list collected from municipal office. From these, 20 old age homes were selected by cluster randomized sampling. Randomization was done using a lottery method. The old age homes list of Delhi is:-

SOUTH

1. Johns Day care and Boarding for Senior Citizen Association
2. Gharaunda
3. Nirmal Chaya
4. Astha Senior Citizen Home
5. Rana old age home
6. Old age home Kalkaji
7. Guru Vishram Vridhashram
8. Har – Mit trust
9. Durga Senior Society
10. Delhi Christian friend in need society

EAST

1. Nirmal Hriday
2. Ashirwad Senior Citizen Council
3. Sukh Dham Old age home
4. Shanti Bhawan, Burari
5. Ozanam Home Society
6. Bhagwadham Dharmarth Varisth Nagrik Aawas
7. St. Marry’s home for aged women
8. Darshan Vishram Vridhashram

NORTH

1. Shri Krishandham Vridhashram.
2. NAB Kaushalya Rani Home for aged blind.
3. Vridh Ashram Triveni Devi Charitable Society
4. Home for aged and infirm person.
5. Kartar Vridh Ghar.
6. Elder home Society.

WEST

1. Sabbarwal Vridhashram
2. Godhuli Senior Citizen home
3. Shri Sukhal Jain Mandir Vridhashram
4. Suhana Savera Old age home
5. Old age home, Tilak Nagar
6. Anand Dham and Vridh Ashram
7. Old age home, Dwarka
8. Old age home, Bindapur
9. Ayudham society for old and infirm.
10. Sewa Salkaip Santham

CENTRAL

1. Sri Geeta Vridhashram
2. Shahid Sainik
3. Guru Nanak Sukhshala
4. Arya Mahila Ashram

The old age homes selected were:-

Guru Nanak Sukhshala, Anand Dham and Vridh Ashram, Godhuli Senior Citizen home, Ayudham society for old and infirm., Vridh Ashram Triveni Devi Charitable Society, Bhagwadham Dharmarth Varisth Nagrik Aawas, St. Marry’s home for aged women, Kartar Vridh Ghar, Elder home Society, Old age home, Tilak Nagar, Anand Dham and Vridh Ashram, Har – Mit trust, Rana old age home, Sabbarwal Vridhashram, Sewa Salkaip Santham, Ozanam Home Society, Old age home, Dwarka, Old age home, Bindapur, Sukh Dham Old age home, Nirmal Hriday

All the inmates were screened for eligibility criteria and a final sample size of 464 subjects was obtained.

### Questionnaire design and validation

Informed consent was obtained from the subjects before starting the examination procedure. The examiner was trained and calibrated prior to the study to ensure uniform interpretation of the codes and criteria for various diseases and conditions to be observed or recorded. The kappa value for all the item was found to be > 0.85. A schedule of the survey for data collection was prepared. On an average 10-15 subjects were interviewed and examined per day.

A survey proforma was prepared based on Oral Health Assessment Form, WHO (2013) [7]. It was used in the present study for assessing oral mucosa .Demographic information was also collected which included name, age, sex, address and religion. Clinical examination was performed using two mouth mirrors under natural illumination. No biopsies or laboratory tests were done in the present study.

According to WHO, a thorough systematic examination was performed in the following sequence: Labial mucosa and labial sulci (upper and lower), followed by labial part of the commissures and buccal mucosa (right and left), Tongue (dorsal and ventral surfaces, margins), floor of the mouth, hard and soft palate, alveolar ridges/gingiva (upper and lower).

### Inclusion Criteria

Those who were 65-74 years old and were residents of these old age homes were included in the study.

### Exclusion criteria

Those who were bedridden, medically compromised and denied the consent for examination were excluded from the study.

### Ethical committee approval

Ethical clearance (2017/20/IEC/MRDC) was obtained from the institutional ethical board. Prior to the study, permission was taken from the concerned authorities of old age homes also after explaining the purpose and procedure of the study.

### Data management and statistical analysis

The data so obtained was compiled systematically. The collected data was processed and analysed by SPSS version 23. Chi square test was used to find the significance between two variables. A p-value less than 0.05 was considered statistically significant (95% confidence interval).

## Results

Out of a total of 464 subjects, 291(62.70%) were males and 173 (37.30%) were females. The mean age was found to be 69. Major ethnic groups was Hindu (81.20%) followed by Muslims (10.60%). Table 1 shows the demographic characteristics of the subjects. Oral mucosal lesions seen in the study subjects were malignant tumour, leukoplakia, lichen planus, ulcerations, ANUG, Abscess and candidiasis. Table 2 shows the oral mucosal lesion prevalence in older individuals residing in old age homes. No statistical difference (p = 0.371) was found among male and female participants when considered for lesion presence and absence.

A malignant tumour was seen in 7 subjects (1.5%) and commonly seen area is floor of mouth. Leukoplakia was seen in 70 subjects (15%) and was present on buccal mucosa in the majority. Lichen planus was also present on buccal mucosa in 19 subjects (4.09%). Ulcerations were commonly seen on the tongue followed by lips i.e. 17 (3.6%) &11 (2.3%) subjects respectively.

Acute necrotizing gingivitis was seen in 13 subjects (2.8%) and candidiasis was seen mostly on the buccal mucosa. The abscess was found mainly on alveolar ridges/gingiva in 23 subjects (4.9%), followed by sulci and hard palate and soft palate. Other conditions were also seen in 7 subjects (1.5%) which include oral sub-mucous fibrosis on the buccal mucosa. Buccal mucosa (19.7%) was the most involved site found in the present study (Table 3).

## Discussion

The overall prevalence of Oral mucosal lesions in elderly residing in old age homes of Delhi was found to be 44%. It is lower than reported among Yemen elder population by Al-Maweri SA, i.e. 77.1% [9]. However, this rate is much higher than the results of other epidemiological studies [10, 11, 12, 13, 14]. Due to variations in social, cultural and demographic of the present study population, the comparison of the results of this study with other studies becomes difficult.

### Distribution of lesion according to Gender

According to gender distribution, Ferriera R C, et al [14] and Rabiei M, et al [15] reported a female preponderance in their cases. However, in the present study, OMLs were more common among men (65%) than women, which is in accordance with several studies [13, 16, 17, 18]. This finding is possibly because men are more exposed to risk habits than are women. Also, men in our community are usually involved in smoking and other risk habits as compared to women. Thus, a higher prevalence of certain lesions among men was not a surprise.

### Leukoplakia

During clinical examination, Leukoplakia (14.7%) was the most commonly observed oral mucosal condition in the study subjects. However, it was less prevalent in the study done by Bansal V, et al i.e. 7.23% [19]. The reason could be that maximum age reported in the study done by Bansal et al was 97 years and it was limited to 74 years in the present study. It has been seen that incidence of OMLs decreases with advancing age due to the fact that older people usually, due to certain medical reasons, quit oral risk habits such as smoking and habitual quid chewing with advancing age. Consequently, the incidence of such lesions will decrease significantly.

### Locations of the lesions

The results of the study done by Bansal V et al (2010) support the present study in the case of most affected site which is buccal mucosa [19]. Further, it was found that commisures were least affected site, followed by vermillion border and hard/soft palate. However, in a study done by Patil S, it is different, i.e. hard palate (23.1%) was the most commonly affected site and soft palate was the least involved (3.6%) [20]. The result of the present study is supported by the study done by Mehrotra R et al, where it was found that both the right and left buccal mucosa were involved in 121(53%) patients.[5] Retromolar trigone was found to be the next most commonly involved area after buccal mucosa. Hard and soft plate, tongue, alveolar mucosa and floor of the mouth were other infrequent sites for oromucosal lesion. [6]

### Abscess and ulceration

The abscess was noted on alveolar ridges and gingiva in 23 subjects, which was due to grossly decayed teeth and root stumps in elderly. Gonsalves W C (2008) [21] discussed that older persons are at risk of chronic diseases of the mouth and found that the most common oral condition in the study population is oral candidiasis and xerostomia (dry mouth). In the present study, the most common oral condition found is leukoplakia followed by ulcerations on the mucosa. Reason for more prevalence of leukoplakia and ulceration in the present study could be that, the homebound elderly does not visit the dentist for a long time which results in deterioration of their oral health. Hence, their mouth becomes more susceptible to these lesions.

### Lichen planus

Lichen planus was seen in 4.4% of the study subjects. This result supports the findings of Mujica [11] and Cebeci [6] studies 3% and 0.8 respectively. Similarly, in the study done by Al-Maweri SA et al, it was observed that lichen planus was seen in 1.6% of study population [9]. But the present study results conflicts with the conclusions of other authors such as Patil S (2015) [20], who found that 18% of the population had lichen planus.

### Malignancies

In the present study, 7 patients (1.4%) were diagnosed as having malignancies, all of whom were diagnosed as having squamous cell carcinoma (SCC). Similarly, in the study done by Pai A [22], it was observed that thirteen patients were diagnosed with squamous cell carcinoma (1.3%). For an oral cancer to develop, mouth is the most susceptible and common location. Most malignancies, especially SCCs involving the mucosal tissue, are usually evident. However, all such potentially malignant lesions should be confirmed by microscopic analysis [11].

### Candidiasis

In the study done by Mujica V et al., in 2008 it was found that only one individual had candidiasis out of 340 institutionalized elderly populations [11]. Similarly, a study done by Pai A [22] reported 5 cases of candidiasis in geriatric population of Bangalore, India. The result of these previous studies supports the present study, as only 4 candidiasis patients were noted in this study. Ulceration was noted in 11.6% of the present study subjects, a percentage comparable to that in a study by Pai A [22], but higher than that of a study done by Cebeci et al. [6]. Reason could be that elders have decreased physical mobility, dependency on help and general tiredness that makes it difficult for them to visit dental clinic even when they have dental problems and it results in their poor oral health.[23, 24, 25, 26].

## Conclusion

As seen in the results of the study, many elders are suffering from various oral mucosal conditions which are worsening their sufferings. They are away from their home due to various reasons but they deserve a better quality of life. Primary care physicians present in old age homes are comparatively in a more frequent contact with the inmates as compared to the dentist. They may play a crucial role in recognizing risk for oromucosal lesions through a focused examination of the oral cavity while performing a general examination and prompt referral to a dentist whenever required.

Further, if a patient is suffering from any physical and mental condition which limits his/her movements, then these patients can get benefit from various dental aids for their oral hygiene maintenance like customized handle brushes, electronic toothbrushes and specially designed floss holders to improve the grip. So, various NGOs and government agencies should come forward for providing these devices to the elder patients in these old age homes.

### Limitation of the study

Limitation of the study was that the various risk habits like smoking, alcohol, lifestyle factors, and oral hygiene practices of the subjects were not asked. As these may be the reason for oral mucosal lesions in elderly.

### Future scope of the study

This study provides epidemiological data on prevalence of oromucosal lesions in elderly residing in old age homes of Delhi, India. It may prove valuable in the planning of future oral health studies in India. Oral mucosal changes are progressive and if not prevented or left untreated, it can affect the general health of the elderly individual. Hence, a periodic and a thorough oral screening and an examination are highly required for the geriatric population of old age homes.

### What is already known on this topic?

To our best knowledge, our study is the one to assess the distribution and prevalence of oral mucosal lesion in the elderly residing in old age homes. Various studies have been done in other parts of the country, but not in Delhi which is national capital of the country.

### What this study adds

Result of the study indicate that there is a need for periodic examination of elder’s oral health, as this will affect their quality of life as well. So, various health promotional programs can be initiated by the government authorities.

## Figures and Tables

**Figure 1:**
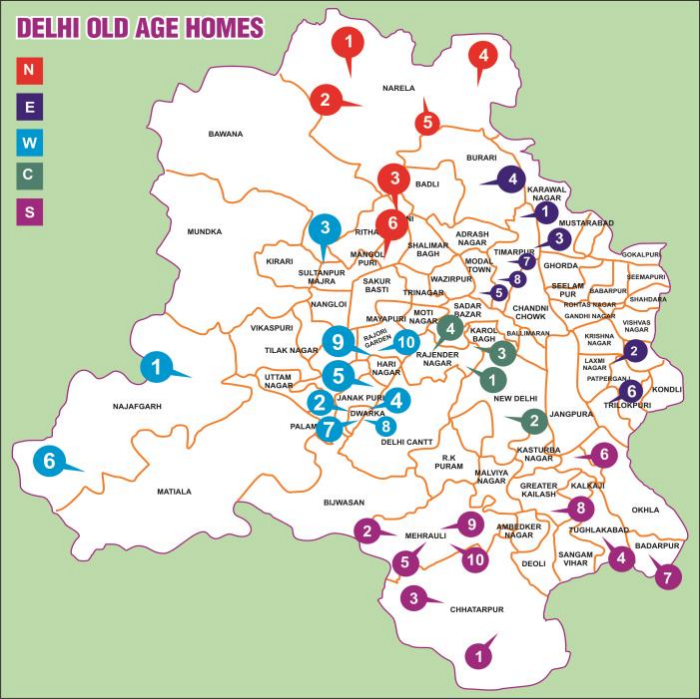
Map of Old Age Homes of Delhi, India

**Table 1:** Demographic characteristic of the study subjects

Variable	Number	Percentage
**Religion**		
Hindu	377	81.20%
Muslims	49	10.60%
Christians	38	8.20%
**Gender**		
Male	291	62.70%
Female	173	37.30%

**Table-2:** Prevalence of oral mucosal conditions in patients

Oral mucosal conditions		Number and percentage of patients with lesion location	Total Number of patients and percentage	95% Confidence interval	
No abnormal condition			260	lower limit	upper limit
**Malignant tumour**	Buccal mucosa	2(28.590%)	7 (1.5%)	0.4	2.7
	Floor of mouth	4 (57.11%)			
	Tongue	1(14.30%)			
**Leukoplakia**	Lips	1(1.40%)	70 (15.0%)	10.2	16.2
	Buccal mucosa	62 (88.60%)			
	Floor of mouth	3 (4.30%)			
	Tongue	4 (6.70%)			
**Lichen planus**	Vermilion border	2 (9.50%)	21(4.52%)	2.7	6.6
	Buccal mucosa	19 (90.50%)			
**Ulceration**	Vermilion border	4(7.40%)	54(11.63%)	8.2	13.9
	Commisures	4(7.40%)			
	Lips	11(20.40%)			
	Sulci	6(11.10%)			
	Buccal mucosa	3(5.60%)			
	Floor of mouth	6(11.10%)			
	Tongue	17(31.50%)			
	Hard/soft palate	3(5.50%)			
**Acute Necrotizing Ulcerative Gingivitis**	Alveolar ridges and gingiva	13(100%)	13(2.80%)	1.5	4.4
**Candidiasis**	Buccal mucosa	4(100%)	4(0.86%)	0.2	1.8
**Abscess**	Sulci	3(10.70%)	28(6.03%)	3.1	6.9
	Hard/soft palate	2(7.10%)			
	Alveolar ridges and gingival	23(82.20%)			
**Other conditions**	Sulci	1(14.30%)	7(1.50%)	0.2	2.2
	Buccal mucosa	4(57.10%)			
	Floor of mouth	1(14.30%)			
	Hard/soft palate	1(14.30%)			

**Table-3:** Site distribution of oral mucosal lesions

Oral mucosal lesion location	Number	Percentage	Confidence interval	
			Lower Limit	Upper Limit
Vermilion border	6	1.29%	0.4	2.2
Commisures	4	0.86%	0.2	2.0
Lips	12	2.58%	1.0	4.0
Sulci	10	2.15%	0.9	3.8
Buccal mucosa	94	20.25%	15	22.3
Floor of mouth	14	3.01%	1.5	4.6
Tongue	22	4.74%	2.2	6.0
Hard/soft palate	6	1.29%	0	1.5
Alveolar ridges and gingiva	36	7.75%	4.9	9.3
Not recorded/not present	260	56.03%	55.5	65
